# Mineral Distributions at the Developing Tendon Enthesis

**DOI:** 10.1371/journal.pone.0048630

**Published:** 2012-11-09

**Authors:** Andrea G. Schwartz, Jill D. Pasteris, Guy M. Genin, Tyrone L. Daulton, Stavros Thomopoulos

**Affiliations:** 1 Department of Orthopaedic Surgery, Washington University, St. Louis, Missouri, United States of America; 2 Department of Earth and Planetary Sciences, Washington University, St. Louis, Missouri, United States of America; 3 Department of Mechanical Engineering & Materials Science, Washington University, St. Louis, Missouri, United States of America; 4 Center for Materials Innovation, Washington University, St. Louis, Missouri, United States of America; 5 Department of Physics, Washington University, St. Louis, Missouri, United States of America; University of Notre Dame, United States of America

## Abstract

Tendon attaches to bone across a functionally graded interface, “the enthesis”. A gradient of mineral content is believed to play an important role for dissipation of stress concentrations at mature fibrocartilaginous interfaces. Surgical repair of injured tendon to bone often fails, suggesting that the enthesis does not regenerate in a healing setting. Understanding the development and the micro/nano-meter structure of this unique interface may provide novel insights for the improvement of repair strategies. This study monitored the development of transitional tissue at the murine supraspinatus tendon enthesis, which begins postnatally and is completed by postnatal day 28. The micrometer-scale distribution of mineral across the developing enthesis was studied by X-ray micro-computed tomography and Raman microprobe spectroscopy. Analyzed regions were identified and further studied by histomorphometry. The nanometer-scale distribution of mineral and collagen fibrils at the developing interface was studied using transmission electron microscopy (TEM). A zone (∼20 µm) exhibiting a gradient in mineral relative to collagen was detected at the leading edge of the hard-soft tissue interface as early as postnatal day 7. Nanocharacterization by TEM suggested that this mineral gradient arose from intrinsic surface roughness on the scale of tens of nanometers at the mineralized front. Microcomputed tomography measurements indicated increases in bone mineral density with time. Raman spectroscopy measurements revealed that the mineral-to-collagen ratio on the mineralized side of the interface was constant throughout postnatal development. An increase in the carbonate concentration of the apatite mineral phase over time suggested possible matrix remodeling during postnatal development. Comparison of Raman-based observations of localized mineral content with histomorphological features indicated that development of the graded mineralized interface is linked to endochondral bone formation near the tendon insertion. These conserved and time-varying aspects of interface composition may have important implications for the growth and mechanical stability of the tendon-to-bone attachment throughout development.

## Introduction

Tendons and ligaments attach to bone across transitional tissue interfaces that are several micrometers to millimeters in thickness. The interface, termed the “enthesis”, is classified as either fibrous (e.g., medial collateral ligament to tibia enthesis) or fibrocartilaginous (e.g., supraspinatus tendon to humeral head enthesis) [1], [2], [3]. The fibrocartilaginous enthesis contains a functionally graded transitional tissue, with variations in extracellular matrix structure and composition giving rise to variations in mechanical properties across the interface [4], [5], [6], [7], [8]. Tendon consists primarily of type I collagen with small amounts of decorin and biglycan. Bone consists of heavily mineralized type I collagen. Collagen fibers are well aligned in tendon. However, the collagen fibers become less organized as they insert into the bone [9]. At the tendon enthesis, a fibrocartilaginous transitional zone is present that is rich in type II collagen and aggrecan produced by fibrochondrocytes, which have a rounder morphology compared to spindle-shaped tendon fibroblast cells and are phenotypically similar to chondrocytes. Within the transitional zone of the rat supraspinatus tendon enthesis, an increase in mineral relative to collagen has been observed through the transition from tendon to bone [10]. These variations in structural and compositional properties result in graded mechanical behavior that contributes to an efficient transfer of muscle load from tendon to bone [11], [12], [13]. It is believed that the gradient in mineral content is particularly important for limiting stress concentrations at the mineralized interface.

In an injury-and-repair scenario, the original graded transitional tissue of the fibrocartilaginous insertion is not recreated after the tendon is surgically reattached to bone. Surgical reattachment leads to a more abrupt interface of mechanically inferior and disorganized scar tissue [14], [15]. The loss of a gradual mineral transition likely contributes to the decreased mechanical performance of the load-bearing interface and results in frequent re-ruptures. For example, surgical repair of massive rotator cuff tears, which relies on tendon-to-bone healing for success, has a re-tear rate of up to 94% [16], [17].

In contrast to the scar tissue that results from healing, developmental processes generate an effective fibrocartilaginous attachment. It remains unclear how the activities of cells coordinate during development to give rise to the complex graded structure of the enthesis. Histological studies have indicated that the enthesis develops post-natally in murine shoulders [18]. Whereas previous studies have examined mineral distributions in the adult tendon enthesis [10], developmental patterns of gene and protein expression for the organic matrix components [18], [19], [20], [21], and the mineral content in the developing bovine ligament enthesis [22], localized mineralization patterns in the developing tendon enthesis have not yet been investigated.

The fibrocartilaginous enthesis has many similarities to the structure of the growth plate that is formed during endochondral ossification of bone [1]. Endochondral ossification is the process by which bone forms from a cartilaginous template [23]. Chondrocytes from the precursor template proliferate, synthesize extracellular matrix, and undergo hypertrophy to enlarge the tissue and eventually terminally differentiate, leaving behind a mineralized cartilage matrix. The mineralized cartilage template is invaded by vascular tissue that delivers bone cell precursors to remodel the mineralized matrix into bone. This process leads to specific gradients in cell type and matrix composition, and is tightly regulated by biological signals and influenced by applied loads. Blitz et al. reported similar events at the developing deltoid insertion into the humeral tuberosity [24].

The objective of the current study was to characterize the spatial distribution of mineral across the tendon-to-bone insertions of developing mouse shoulders. Specifically, our goal was to better understand the time sequence and stages of development of the graded region found at the interface between mineralized and non-mineralized fibrocartilage in the enthesis. We hypothesized that a region of graded mineral content would develop concurrently with the appearance of fibrocartilage in the insertion. Surprisingly, our results show that a mineral gradient appears near the enthesis much earlier in development and is associated with the mineralization front. The micrometer- and nanometer-scale distributions of mineral across the attachment have important mechanical implications for the functional behavior of the tissue. Comparing the micrometer-scale patterns of mineralization to cell and matrix morphologies may provide insights into the developmental mechanisms controlling enthesis mineralization. Moreover, understanding the structure of the tendon-to-bone interface throughout maturation at these different spatial hierarchies can assist development of novel biomimetic materials and signaling molecules that can enhance clinical approaches for surgically repairing tendon-to-bone injuries.

## Methods

### Animal Model

The use of animals and our procedures for this study were approved by the animal studies committee at Washington University (Protocol Number: 20100091) and all efforts were made to minimize suffering. CD1 mice (Charles River Labs) were sacrificed in a CO_2_ chamber at five postnatal (P) time points (days): P7, P10, P14, P28, and P56. The time points were chosen to coincide with epiphyseal mineralization, which begins ∼P3–P7 in the mouse shoulder, and the appearance of mature fibrocartilage at the insertion, which appears ∼P21–P28 [18], [19], [25]. Five animals per time point were allocated for Raman analysis and subsequent frozen section histology, three animals per time point were scanned for X-ray microcomputed tomography and processed for paraffin histology, and two animals per time point were allocated for TEM analysis. The animal sex and the choice of right or left shoulder were randomized for each time point and assay. Detailed descriptions of the specimen preparation procedures are described below.

### Raman Microprobe Analysis

Raman spectroscopy was used to probe micrometer-scale variations in relative mineral concentration across the tendon-to-bone interface. This technique can be used to study unfixed, hydrated tissues, offering significant advantages over other micrometer-scale compositional analysis methods [10], [26]. Animals allocated for Raman analysis (N = 5 per time point) were stored at −20°C. After thawing, the humeral head and supraspinatus tendon were isolated, taking care to preserve the tendon-to-bone insertion. The samples were then embedded in optimal cutting temperature (OCT) embedding medium without further processing and re-frozen at −80°C. 20 µm-thick sections of fresh un-decalcified tissue were cut in the coronal oblique plane on a cryostat. Sections were placed on glass slides and stored at −80°C. A recent study indicates that multiple freeze-thaw cycles of bone tissues may alter some portions of the Raman spectrum [27], [28]. However, for our evaluation of the degree of mineralization, we did not rely on the amide I and amide III bands, whose stability during freeze-thaw cycles was called into question in the prior study. Immediately prior to analysis, sections were washed three times with phosphate buffered saline (PBS) to thaw the samples and remove remaining embedding medium and any blood contamination from the bone marrow.

The Raman microprobe apparatus (HoloLab Series 5000 fiber-optically coupled Raman Microscope, Kaiser Optical Systems, Inc.) and analysis procedures have been described previously [10]. Briefly, Raman spectra were collected using 10 mW (measured at the sample surface) of 532 nm laser excitation focused to an approximately 1 µm beam spot on the surface of the specimen using an 80× objective lens with a N.A. = 0.85. Lateral and depth resolution during sample analysis were on the order of a few micrometers. Although the 532 nm laser line used for this study allows for excellent signal-to-noise at relatively low laser power, blood contamination can present a problem, as it introduces a high fluorescence background under 532 nm excitation. Fortunately for our study, the tendon-to-bone insertion is relatively avascular, and any residual red blood cells from the bone marrow were removed by rinsing with PBS. The light scattered by the illuminated specimen was collected in a backscattered configuration through the same objective lens used to focus the laser onto the specimen. A 2048-channel CCD detector was used to concurrently detect the spectral range 100–4000 Δcm^−1^. Each spectrum used for analysis was the average of 32, 4-second spectral acquisitions. The laser spot was manually focused onto the surface of each sample using reflected light images as a guide to the surface topography.

**Figure 1:**
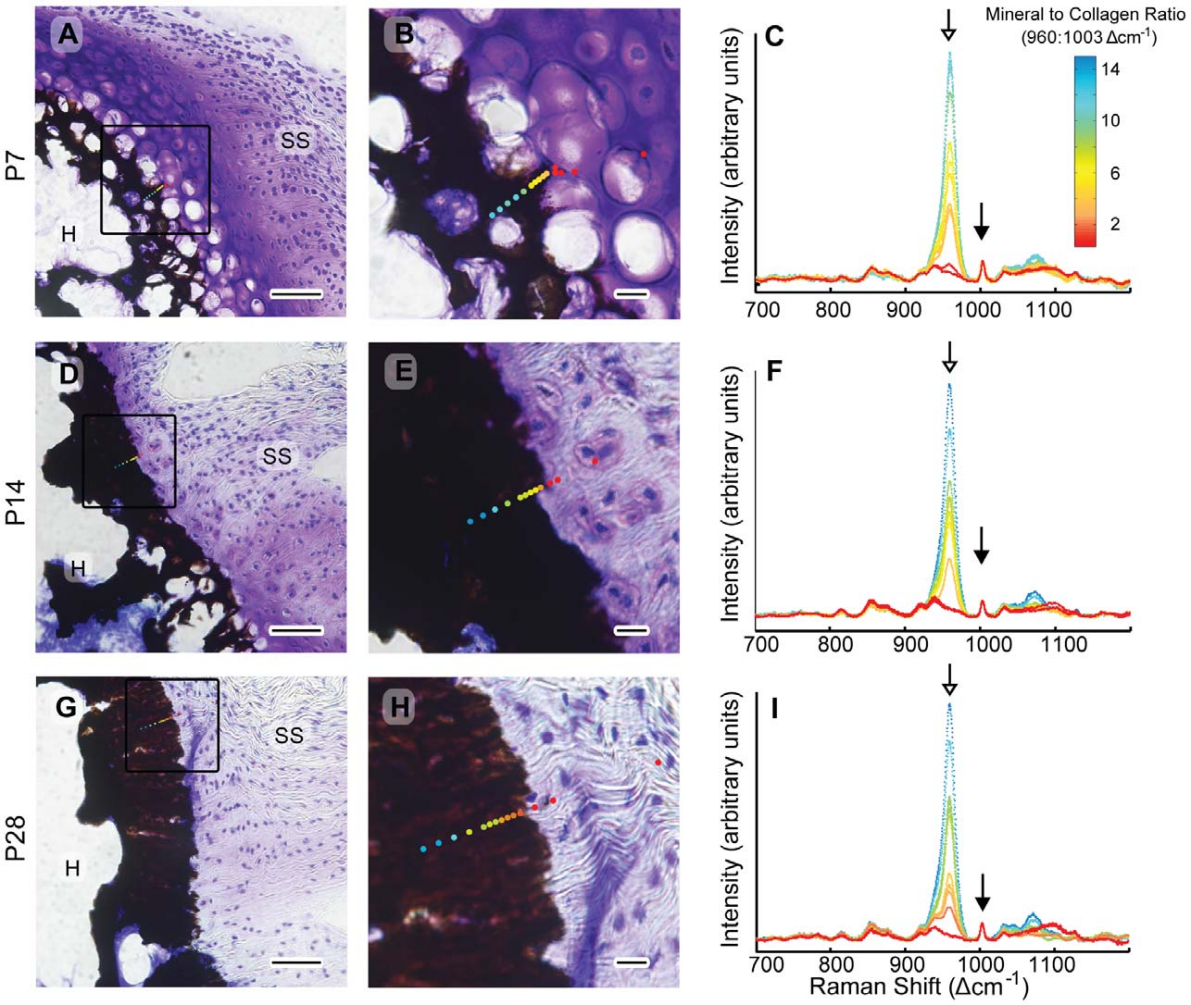
Raman microprobe analysis of developing supraspinatus tendon insertions. Top row (A–C) shows a P7 insertion, middle row (D–F) a P14 insertion, and bottom row (G–I) a P28 insertion. Left column, A, D, G: 20 µm-thick sections stained with toluidine blue and according to von Kossa's method (scale bar = 50 µm). Note that despite the sharp front of mineralization in these figures suggested by the von Kossa staining, a graded mineralization from is evident from the Raman scans. Middle column B, E, H: magnified view of square region of interest shown in images in the left column (scale bar = 10 µm). The relative mineral concentration determined by the ratio of the heights of the 960 Δcm^−1^ to 1003 Δcm^−1^ Raman peaks (corresponding to the ν1 P-O stretching band of hydroxylapatite and the aromatic ring stretching band of phenylalanine in collagen, respectively) is indicated by the color gradient of the overlaid points. SS: supraspinatus tendon and H: humeral head. Right column C, F, I: baseline-corrected Raman spectra corresponding to points along a traverse from tendon (no mineral - dark blue) to bone (high mineral - red). The mineral peak (960 Δcm^−1^) is indicated with a hollow arrow and the collagen peak (1003 Δcm^−1^) is indicated with a black-filled arrow.

The hydrated state and dramatically different material properties between tendon and bone made it impossible to create a polished surface that was flat enough for extensive 2D automated mapping. Instead, for each sample, spectra were acquired from at least 12 different regions along a roughly linear transect that spanned the tendon-to-bone interface. Each region of interest was manually selected to optimize the Raman signal. Example traverses are shown in Figure 1. The x-y stage position of the region of interest corresponding to each acquired spectrum was documented on the reflected light images acquired before each spectrum. The acquired Raman spectra were analyzed to interpret compositional properties of the tissue at each location. This study was primarily concerned with the degree of mineralization (i.e., mineral-to-matrix ratio) and the spatial distribution of mineral in the tissue. In the non-mineralized tissue, no spectral differences were observed between fibrocartilage and tendon. The fully mineralized side of the insertion was defined where a plateau occurred in the spectroscopically determined relative mineral concentration in the tissue. To ensure that the traverses accurately captured the typical characteristics of the interface, three traverses were collected at different locations along the width of the insertion for several samples, and it was determined that the gradient region was consistent across all regions analyzed (data not shown). These pilot data indicated that the gradient was maintained along the width of the insertion and a single traverse was representative of the gradient..

### Raman Spectral Analysis

Raman spectra were background corrected using the software package Grams32 (Galactic, Salem, NH) via a linear curve-fitting operation on approximately 10 operator-defined points. The relative mineral concentration at each region of interest was inferred from the ratio of the intensities of the peaks at 960 Δcm^−1^ and 1003 Δcm^−1^ for the ν_1_ P-O stretching band of hydroxylapatite and the aromatic ring stretching band of phenylalanine residues in collagen, respectively. Many different reference peaks for collagen have been previously used, including the non-specific C-H stretching band (2940 Δcm^−1^) [10], [29], the amide 1 band (1667 Δcm^−1^) [30], [31], and the proline/hydroxyproline bands (855 Δcm^−1^ and 921 Δcm^−1^) [32]. Here, the 1003 Δcm^−1^ peak was chosen as a reference due to its proximity to the P-O stretch in hydroxylapatite at 960 Δcm^−1^. Fluorescence contributions to the background vary widely across the full spectral range and by selecting peaks in close proximity, their background intensities should be nearly identical. Additionally, the peak at 1003 Δcm^−1^ does not overlap other nearby peaks that might confound the measured intensity. In order to validate this reference peak, additional analyses were performed using alternate collagen peaks (2940 Δcm^−1^ and 1667 Δcm^−1^) for reference (data not shown), and the overall trends in the data were unaffected. The analytical value of using the 1003 Δcm^−1^ peak is also supported by our previous work [10]. It is important to recognize that, due to the nature of Raman back-scatter spectroscopy, the ratio of mineral signal strength divided by collagen signal strength does not indicate the absolute volumetric or mass ratio of mineral content to collagen content. However, changes in the spectral ratio have unambiguous meaning. Increases in this spectral ratio indeed do indeed reflect increases in relative mineral concentration in the tissue but do not account for changes in the total amount of extracellular matrix.

To analyze changes in the properties of fully mineralized fibrocartilage near the interface as a function of age, spectra from the fully mineralized side of the insertion underwent further analysis for each sample. Three regions from each sample were identified as areas along the traverse where the mineral-to-matrix spectral ratio had reached a roughly constant maximum value. The traverses were ended before reaching the edge of the marrow cavity underlying the insertion tissue in order to exclude trabecular bone from this analysis. Therefore, the average spectra can be used to gauge the maximum relative mineral concentration of the enthesis fibrocartilage away from the interface. The three measurements from each tissue sample were averaged before statistical comparisons were made between time points. Spectra were background corrected, and peaks within the spectral region of interest were deconvolved from each other based on a mixed Gaussian-Lorentzian algorithm within the GRAMS32 software. The full width at half maximum of the 960 Δcm^−1^ peak (attributed to hydroxylapatite) was used as a measure of crystallographic atomic order, since narrower peaks suggest less structural variation in bond distances and angles. The carbonate content of the mineral crystallites was evaluated by taking the ratio of the heights and the ratio of the areas of the 1070 Δcm^−1^ peak (indicating carbonate substitution for phosphate) against the 960 Δcm^−1^ peak. For spectra with lower signal∶noise ratios, we previously determined that ratios of peak heights are more reproducible than that of peak areas [10]. The 960 Δcm^−1^: 1003 Δcm^−1^ peak height ratio was evaluated to determine the maximum relative degree of mineralization of the tissue.

### Histology and Image Analysis

After Raman analysis, sections were fixed in 10% neutral buffered formalin and stained using von Kossa's method (to visualize the mineral) and toluidine blue (to visualize the cells and matrix). A custom MATLAB (2009a, MathWorks, Natick, MA) script was written using operator-defined control points to align the reflected-light images obtained during spectroscopic analysis with the tissue features visible in the corresponding histology image. This image correlation assured that the regions of interest analyzed by Raman spectroscopy would be associated with their respective histological features with an accuracy of a few micrometers. Several examples of the specific locations of the Raman analyses are overlaid onto histology images, which are presented together with their respective spectra in Figure 1. To compare the position of the mineralization front between age groups, the von Kossa- and toluidine blue-stained images were used to estimate the average distance between the stained mineral front and the tendon proper. The location of tendon tissue was approximated by the location of stained tendon fibroblasts, as distinguished by cell morphology and matrix relative to the fibrochondrocytes and proteoglycan-rich stained regions. The position of each acquired Raman-analyzed region of interest was determined relative to the von Kossa-stained mineral front and the tendon boundary. The slope of the mineral gradient was calculated for each animal by plotting the Raman-measured mineral-to-matrix ratio versus the radial distance to the edge of the von Kossa-stained mineral front and performing a linear regression using a window of at least 6 sequential points. That window was rejected if the least squares fit was not statistically significant (p<0.05). The slope was then defined as the first-order term calculated from the window of points that resulted in the greatest r^2^ statistic. This analysis resulted in an average r^2^ statistic for all of the samples of 0.91±0.08.

Additional 5 µm-thick sections from decalcified, paraffin-embedded specimens (N = 3 per time point) were stained with toluidine blue and used to measure cell areas relative to extracellular matrix at each time point. Mineralized areas of the insertion were identified by close proximity to the bone side of the insertion and comparison to mineralized stained sections. Image analysis was performed using ImageJ (NIH, Bethesda, MD).

### Microcomputed Tomography

Three freshly dissected samples from each time point were X-ray scanned for micro-computed tomography (μCT40; Scanco Medical AG, Switzerland). The X-ray tube settings were 55 kV and 145 uA and the integration time used was 99 ms. The resulting reconstructed isometric voxel size was 30 µm (i.e., each voxel consisted of a volume of 2.7×10^4^ µm^3^). The humerus was potted in agarose and scanned with the muscle, tendon, and humeral head suspended in air. Using this method, the tendon was easily identified in the reconstructed images. By comparison to hydroxylapatite standards, bone mineral density was calculated from manually selected regions within the reconstructed three-dimensional x-ray data sets encompassing the mineralized fibrocartilage within the tendon insertion. The analyzed regions were selected by identifying only the mineralized regions directly adjacent to the tendon and excluding the surrounding bone. The average total analysis volume for each sample was ∼0.1 mm^3^.

### Transmission Electron Microscopy (TEM)

For TEM nano-characterization, the humerus with attached supraspinatus tendon was micro-dissected to isolate the insertion with a small piece of underlying bone attached (N = 2 per time point). Samples were fixed in 2% paraformaldehyde and 2.5% glutaraldehyde in 0.1 M cacodylate buffer, post fixed with osmium tetroxide, dehydrated in graded ethanol and embedded in Eponate 12 resin. Ultrathin sections (∼70 nm thickness) were sliced on a Leica EM UC6 ultramicrotome. Aqueous processing (and chemical fixation) of bone have been reported to induce structural modification to collagen fibrils as well as to induce some phase transformation, dissolution, and re-precipitation of bioapatite [33], [34]. Therefore, the boat of the diamond knife was filled with ethylene glycol rather than water in order to limit aqueous dissolution of the mineral phase. Sections were mounted on Cu TEM grids either uncoated or coated with amorphous carbon. Bright-field TEM images of unstained sections were collected using a Hitachi H 7500 transmission electron microscope operating at 80 kV equipped with a CCD camera.

One sample from the P56 time point was nanocharacterized using a JEOL JEM-2100F field-emission scanning transmission electron microscope (for an instrument description, see [35]). Shortly after insertion into the scanning (S)-TEM instrument column, the specimen was subjected to a >1 hr beam shower to fix any hydrocarbons (and prevent spot contamination) prior to exposing the specimen to a focused ∼1 nm FWHM diameter probe. Specimens were imaged in STEM mode using the high-angle annular dark-field (HAADF) detector, which can measure electrons at high scattering angles (in our case, the range 36.8±0.1 to 128.0±0.4 mrad) where scattering is approximately incoherent. The high-angle scattering cross section for the volume probed by the electron beam is approximately proportional to the square of the mean atomic number in that volume. Therefore, high-angle scattering from heavy atoms will be more intense than from light atoms, producing an STEM-HAADF image with atomic *z*-contrast. Specimens were also spectrally imaged using a GIF Tridiem capable of electron energy loss spectroscopy (EELS). At each image pixel position a core-loss spectrum was collected over a 409.6 eV range starting at ∼232 eV. The following conditions were used during collection of STEM-EELS spectral images: a collection angle of 2*β* = 22.66±0.06 mrad, a 5 mm diameter spectrometer entrance aperture, and an energy dispersion of 0.2 eV/channel. Spectra were corrected for dark current and channel-to-channel gain variation of the GIF CCD detector array and collected in the diffraction mode of the transmission electron microscope (i.e., image coupling to the EELS spectrometer). The spectra were processed using Gatan Digital Micrograph by fitting a power-law background to each of the pre-edge regions. The background was subtracted from each edge signal, which was then integrated over a 45 eV wide window. Ratios of integrated EELS core-loss signal between two elements were converted to their corresponding atomic ratios using partial cross-sections that were calculated from theoretical Hartree-Slater models. Unlike maps of EELS core-loss signal, maps of relative elemental compositions are not influenced by variations in specimen thickness and electron diffraction [36].

### Statistical Analysis

An analysis of variance (ANOVA) was applied to histomorphology measurements (matrix-to-cell area ratio), Raman measurements (gradient slope, peak height ratios, and peak widths), and X-ray microcomputed tomography measurements (bone mineral density) for the factor animal age. When the ANOVA was significant, a post hoc analysis was performed using Fisher's least significant differences tests. Significance was defined as *p*<0.05, and data are reported as the mean (error bars represent the standard deviation of the mean). Significant differences between time points are indicated above each bar.

## Results

### Microscale mineral gradients at the developing enthesis

Raman microprobe analysis demonstrated an increase in mineral content across the murine supraspinatus tendon-to-bone interface at all time points studied. Following Raman analysis, tissue sections were stained for histological analysis. The Raman measured regions of interest (documented by light microscopy images), were registered to and overlaid onto the subsequently collected histological images (Figure 1). Mineral was first detected by Raman at the edge of the von Kossa stained mineralization front. The ν_1_ P-O stretching band of hydroxylapatite was absent on the tendon side of the von Kossa stained mineralization front. The image at the interface produced by von Kossa staining was either transparent or opaque (with little gradation), implying an abrupt interface between mineralized and non-mineralized tissue at the resolution of the optical microscope. In contrast, the Raman data revealed a wider region of graded mineral-to-collagen ratio over a scale of ∼20 µm. These seemingly contradictory results highlight the difference in sensitivity of the two techniques to variations in mineral content (i.e., low sensitivity of von Kossa staining and high sensitivity of Raman spectroscopy). While von Kossa staining is excellent for producing a strong signal for a low volume fraction of mineral, it is relatively ineffective at distinguishing densely mineralized regions from sparsely mineralized regions.

**Figure 2:**
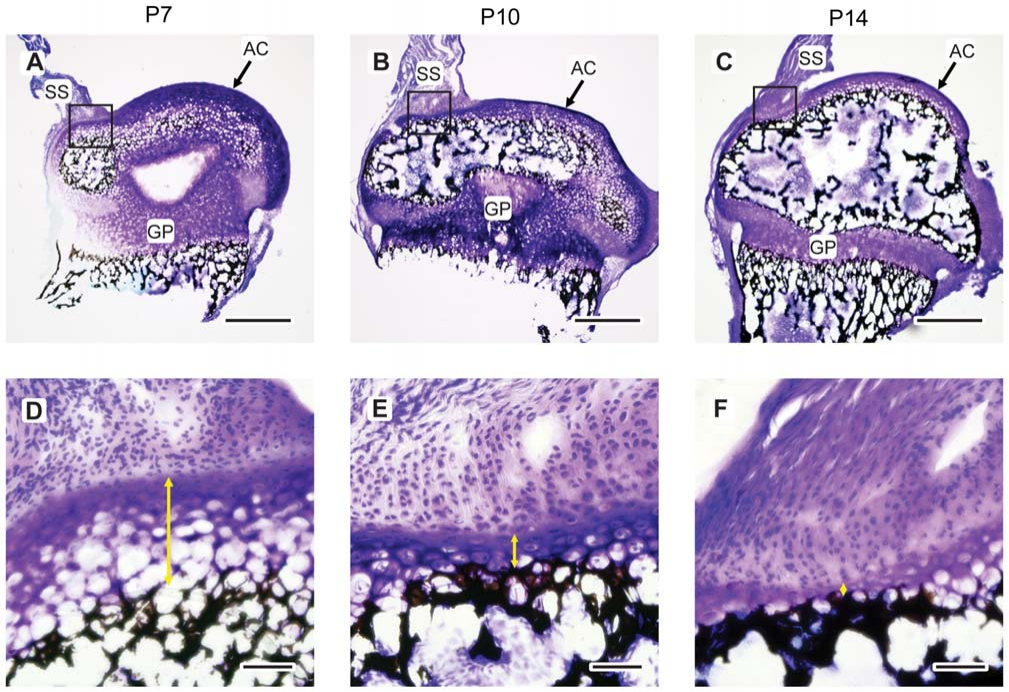
Postnatal mineralization of the humeral head. Top row A, B, C: 20 µm-thick sections of toluidine blue- and von Kossa-stained mouse humeral heads with attached supraspinatus tendons at P7, P10, and P14 (scale bar = 500 µm). Bottom row D, E, F: Magnified views of the square regions of interest shown in the images of the top row (scale bar = 100 µm). Yellow arrows indicate the distance from the mineralization front to the tendon proper. SS: supraspinatus tendon, AC: articular cartilage, GP: growth plate. Top scale bars are 500 µm and bottom scale bars are 100 µm.

The developmental origin of the mineral gradient was investigated by examining shoulders at P7 through P56. Raman traverses indicated a gradient in mineral concentration in the mineralizing cartilage nearest to the insertion as early as P7. Reference to histomorphological features demonstrated that the location of Raman detected mineral coincided with the leading edge of the secondary ossification center closest to the supraspinatus insertion (Figure 2). The observed increase in mineral concentration from the non-mineralized side to the mineralized side of the insertion occurred in narrow (∼10 µm) regions of extra-cellular matrix located between hypertrophic chondrocytes. A similar result was observed for the P10 specimens. At all time points beyond P28, the morphology of the insertion was consistent with mature insertions; fibrochondrocytes were much smaller in size compared to earlier time points, and there was no evidence of epiphyseal cartilage between the mineral front and the tendon proper. At the P14 time point, the specimens exhibited either of the two morphologies described above.

**Figure 3:**
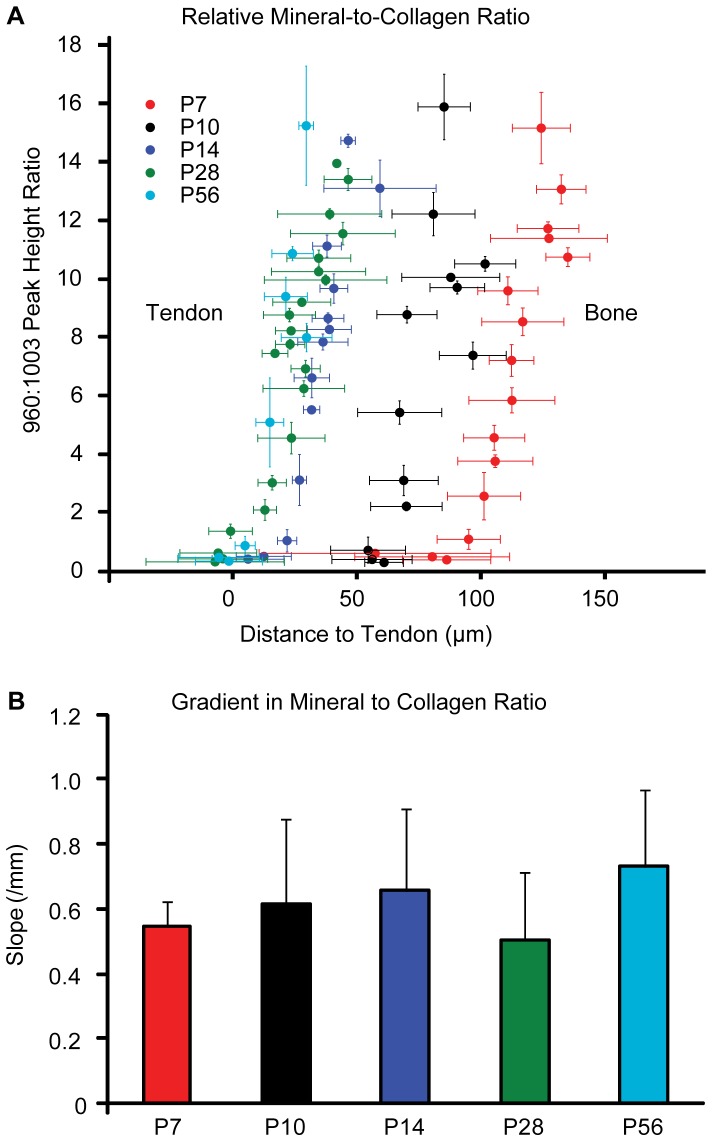
Quantitative analysis of mineral gradients in the developing enthesis. A) The average mineral relative to collagen concentration as gauged by the ratio of the heights of the 960 Δcm^−1^ to 1003 Δcm^−1^ Raman peaks (corresponding to the ν_1_ P-O stretching band of hydroxylapatite and the aromatic ring stretching band of phenylalanine in collagen, respectively) plotted vs. the location relative to the tendon for the different time points in this study (P7, P10, P14, P28, and P56). The data plotted represent the mean of 5 separate measurements (i.e., from 5 mice) and the error bars represent the standard deviation of the mean. B) The slope of the data plotted in A (representing the magnitude of the gradient in mineral content) is shown. The slope was calculated from a linear least squares fit to the data from each animal individually (shown together in A) and the error bars represent the standard deviation of the slope fitting parameter across 5 animals per time point. The magnitude of the mineral gradient remains constant within measurement error throughout the postnatal development time points examined.

In order to quantify micrometer-scale patterns of mineralization in the gradient region, the magnitudes of the mineral gradients (i.e., the slopes of mineral concentration vs. spatial position) were determined for all the time points. The position of each analysis region relative to the tangent to the mineral boundary (as determined by von Kossa staining) was determined. The rate of increase in mineral concentration with position along the insertion from the non-mineralized to the mineralized side did not vary within measurement error across the five post-natal time points investigated (Figure 3A and 3B). The width of the zone of increasing degree of mineralization (i.e., the gradient) also remained approximately constant at about 25 µm from P7 to P56 (Fig. 3A). This width was calculated by dividing the maximum degree of relative mineralization for each sample by the gradient region slope (determined from a linear least squares fit of the mineral-to-matrix spectral ratio versus the absolute distance to the histological appearance of tendon).

### Characteristics of mineralized collagen at the insertion

**Figure 4:**
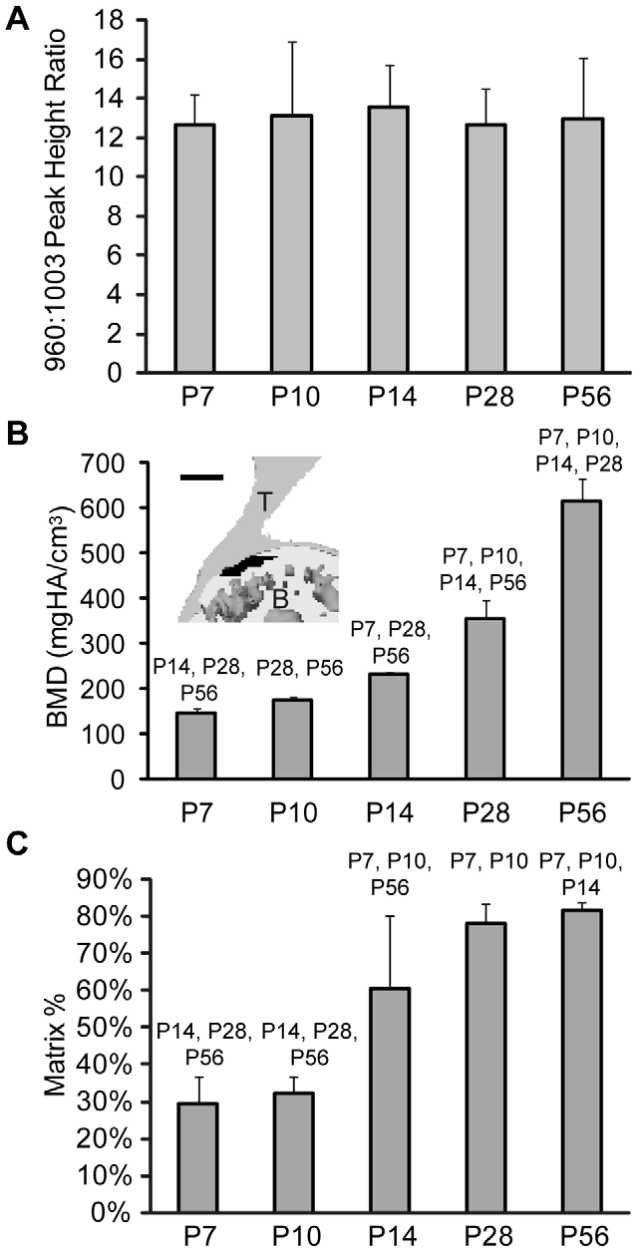
Analysis of mineral content in the developing insertion. A) The average mineral concentration of fully mineralized fibrocartilage was determined by the ratio of the heights of the 960 Δcm^−1^ to 1003 Δcm^−1^ Raman peaks for the different time points in this study (P7, P10, P14, P28, and P56). The average was taken over 3 regions from each traverse that were within the fully mineralized fibrocartilage closest to the bone side of the insertion. The data plotted represent the mean of 5 separate measurements (i.e., from 5 mice) and the error bars represent the standard deviation of the mean. B) Bone mineral density (BMD) measured by X-ray microcomputed tomography increased throughout postnatal development in the mineralized region underlying the supraspinatus tendon (p<0.0001). The numbers above the bars indicate p<0.05 relative to the indicated time point. The inset shows a representative analysis region in black (scale = 500 µm). C) Area fraction of matrix relative to cells, as measured from histological sections, increased throughout postnatal development in the mineralized areas of the insertion (p<0.001). Significant differences between timepoints are indicated above each bar.

To further characterize the accumulation of mineralized tissue at the insertion, three measures of mineral were used: 1) the ratio of collagen (1003 Δcm^−1^) to mineral (960 Δcm^−1^) Raman peak heights, 2) the matrix area fraction, as determined by histomorphometry, and 3) mineral density of bone, as determined by X-ray microcomputed tomography. The Raman-measured mineral-to-collagen spectral ratio from fully mineralized tissue underlying the insertion did not change with animal age (Figure 4A). The value for fully mineralized tissue was based on the mean of three spectra from ∼1 µm in diameter regions of interest (∼1 µm depth penetration) that spanned the mineralized tissue layer underlying the insertion. X-ray microcomputed tomography measurements of the density of the same region of mineralized tissue that underlies the supraspinatus tendon insertion (but over a larger area) indicated increasing bone mineral density over the course of postnatal development (Figure 4B and inset). Bone density was determined from averaging X-ray intensity from ∼30 µm isometric voxels (each with a volume of 2.7×10^4^ µm^3^) within a 0.1 mm^3^ volume encompassing the mineralized portion of the insertion. An example of a typical analysis region is shown in the inset to Figure 4B.

While the ∼1 µm^3^ volumes analyzed by Raman excludes cells, the larger volume analyzed by X-ray micro-computed tomography includes cells. Therefore, we quantified the histologically observed change in overall cell area by measuring cell areas as a fraction of total area (including cells and matrix) in regions corresponding to mineralized insertion transitional tissue in histologic images. In mineralized regions, cell area decreased as postnatal time points advanced (Figure 4C).

**Figure 5:**
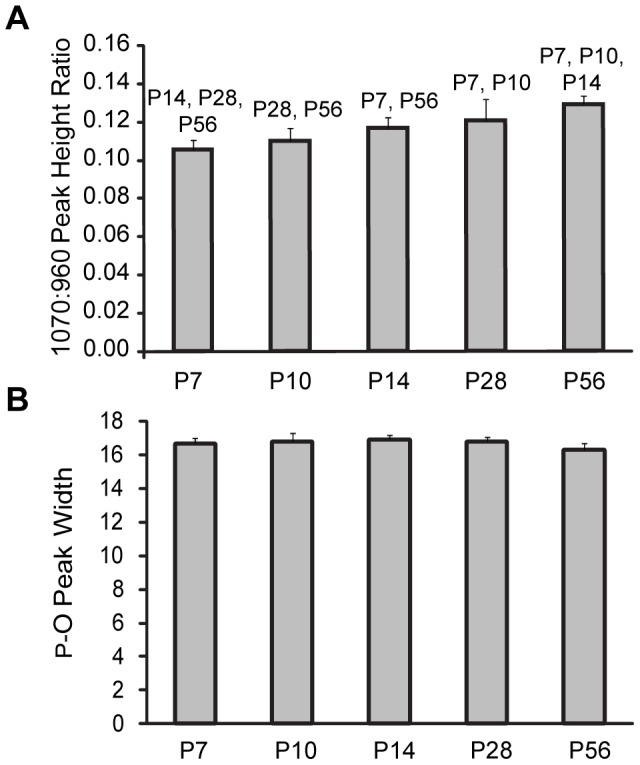
Raman analysis of the matrix mineral suggests remodeling. A) The average degree of carbonate substitution in apatite, as gauged by the ratio of the heights of the 1070 Δcm^−1^ to 960 Δcm^−1^ Raman peaks (corresponding to the ν_3_ PO_4_^3−^ and ν_1_ CO_3_^2−^ stretching bands of carbonate and the ν_1_ P-O stretching band of hydroxylapatite, respectively), within the fully mineralized insertion fibrocartilage increased with age (p<0.001). B) The atomic order in the apatite crystallites, as gauged by the peak width of ν_1_ P-O stretch at 960 Δcm^−1^ (the more narrow the peak, the more crystalline), did not change over time outside of measurement error. For A and B, the average was taken over the 3 regions of interest from each traverse within the fully mineralized fibrocartilage close to the bone side of the insertion. The data plotted represent the mean of 5 separate measurements (i.e., from 5 mice) and the error bars represent the standard deviation of the mean. Significant differences (p<0.05) between time points are indicated above each bar in A.

Raman spectroscopic analysis was used to investigate characteristics of the mineral crystallites within fully mineralized tissue adjacent to the mineral gradient region. Two parameters were measured based on the three analysis regions closest to the mineralized side of the insertion: 1) carbonate concentration of apatite mineral and 2) relative atomic order in the lattice of the mineral crystallites (i.e, crystallinity). Changes in these measures may be indicators of mineral age and remodeling [30], [31], [37]. The extent of carbonate substitution for phosphate was measured as the ratio of the heights of the 1070 Δcm^−1^ peak over the 960 Δcm^−1^ peak for carbonate and phosphate, respectively [37], [38]. Carbonate concentration increased significantly with animal age across the five developmental time-points investigated (Figure 5A). The change in peak height ratios of 0.11 (P7) to 0.13 (P56) with respective peak area ratios of 0.14 and 0.17 corresponds to an increase in carbonate concentration of ∼5.0 wt.% to 5.8 wt.% [37]. Relative atomic order within the lattice of the apatite nanocrystals, one aspect of crystallinity, was evaluated based on the full width at half maximum of the ν_1_ phosphate band at 960 Δcm^−1^. There were no statistically significant differences in crystallinity as a function of animal age (Figure 5B).

### Nanoscale characterization of the mineralized interface

**Figure 6:**
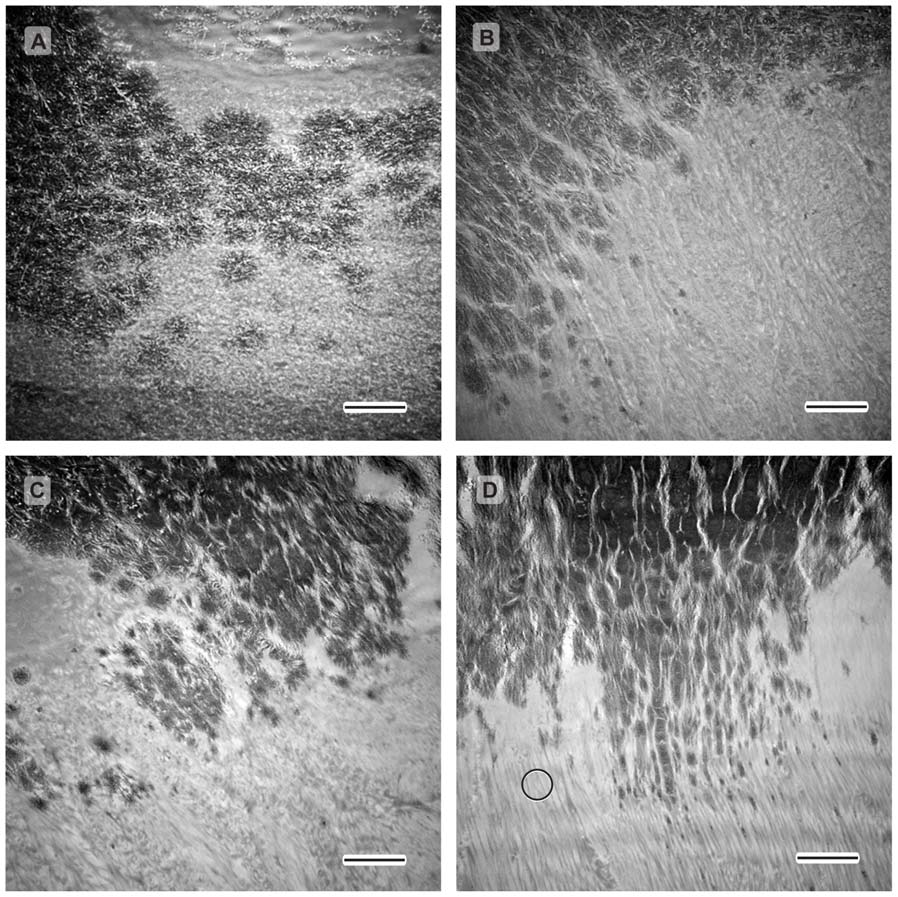
TEM images of the mineralized interface in the supraspinatus tendon enthesis. Bright-field images for various postnatal development times: A) P10, B) P14, C) P28, D) P56 (scale bar = 2 µm). Dark areas indicate mineral (e.g., see Figure 7). The white circle in D shows the approximate probe diameter of the laser used for Raman spectroscopy in this study.

**Figure 7:**
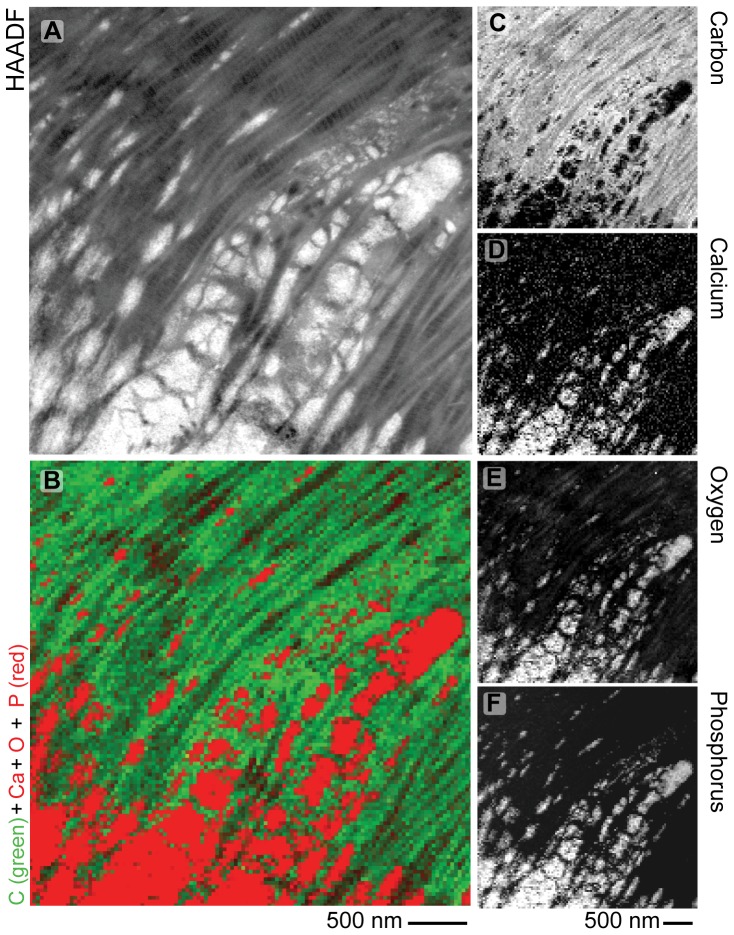
Mineralized interface observed at the supraspinatus tendon-to-bone insertion 56 days after birth. The interface is not smooth and planar but rather exhibits significant “surface roughness” at the length scale of tens of nanometers. A) STEM- high angle annular dark field (HAADF) image. Brighter STEM-HAADF image intensity in regions of uniform thickness corresponds to higher mean atomic number (i.e., locations of mineral relative to collagen). B) STEM-electron energy loss spectroscopy (EELS) spectral image map of C (in atomic (at) % shown by green intensity) as well as summed Ca, O, and P (shown in at% shown by red intensity). Brighter color indicates higher concentrations. STEM-EELS mapped elemental composition (in at%) for C) carbon, D) calcium, E) oxygen, and F) phosphorous. While all tissues will contain calcium, oxygen, and phosphorus to varying amounts, the major sources of these signals are spatially well correlated to one another as well as correlated to the STEM-HAADF intensity and are indicative of apatie. The gray-scale ranges for the STEM-EELS maps are not normalized between 0 at% and 100 at% but rather have been independently adjusted to enhance visual clarity (consequently they are not directly comparable).

In order to further investigate the gradient in relative mineral concentration determined by Raman spectroscopy (Figure 1), the nanostructure of the interface was characterized by TEM. Bright-field TEM images (Figure 6) exhibit regions of dark image contrast. Similar regions in a P56 specimen were found to be rich in calcium, oxygen and phosphorus (i.e. mineral) by STEM-EELS (Figure 7), and this likely holds true for the other time points examined. In bright-field TEM images of the mineralized interface, clusters of mineral (i.e., regions of dark contrast) were observed that increased in size and density along the insertion (Figure 6). Before P14, the matrix appeared disorganized. It contained roughly oval, dendrite-like clusters of mineral approximately 1 µm in diameter. The density of these clusters increased across the mineralized interface from tendon to bone. At P14, similar large clusters of mineral were observed on the humeral head side of the interface, but some appear to be patterned by the increasingly ordered larger collagen fibrils. By P28, the mineralization morphology appeared to be driven by the structure of larger, aligned collagen fibrils at the edge of the interface. Furthermore, elemental mapping indicated that in regions near the interface, mineral was confined to discrete patches associated with the collagen fibrils. There was no evidence of homogenously dispersed mineral crystals or mineral clusters in collagen-rich regions.

## Discussion

This study investigated the development of the mineralized interface at the tendon-to-bone insertion. The aim of this study was to understand the developmental origin of the mineral gradient identified at the mature tendon-to-bone interface. This region is believed to play a critical role in the dissipation of stresses at the tendon-to-bone interface, thereby preventing ruptures at the interface [11], [13]. Previous analyses of this region have indicated an increase in mineral content near the insertion as the humeral head mineralizes during postnatal development [22], [39]. Microcomputed tomographic measurements in the current study similarly showed mineral density increased throughout postnatal development. At a higher spatial resolution, Raman microprobe analysis revealed the presence of a mineral gradient near the insertion early in postnatal development–a gradient that persisted through P56. Raman spectroscopic analysis also showed a relatively constant mineral-to-collagen ratio among the fully mineralized regions at all time points studied. Further investigation of the mineralized interface by TEM at nanoscale resolution demonstrated a high density of mineral clusters that were discontinuous and exhibited a finger-like morphology that protruded perpendicular to the region of continuous mineralization. Collagen fibrils appeared to extend continuously from the tendon region, through the discontinuous mineralized front, into the heavily mineralized region.

We identified the gradient region using Raman analysis and compared its location to histological features in order to characterize the formation of this interface. Fibrocartilage tissue, a critical component of the transitional tissue between tendon and bone, is not histologically evident at the insertion until 3–4 weeks after birth [18]. In contrast to this timeframe, a gradient in mineral content appears near the insertion as early as P7. The current study further indicated that, instead of co-developing with fibrocartilage, the mineral gradient is intrinsic to mineralization fronts associated with endochondral ossification. This association is consistent with the observation that the mature enthesis is similar to the developing growth plate and has characteristics of an arrested growth front [1], [24]. In the humeral head, the secondary ossification center drives mineralization of the cartilaginous bone template over the first two weeks of postnatal development. The mineralization front gradually expands outward as chondrocytes of the cartilage template are terminally differentiated and the matrix mineralizes [23]. Our results indicate that the mineralization front is adjacent to the developing tendon by P14. Prior to this time, the tendon inserts into the epiphyseal cartilage of the humeral head, which is not yet mineralized. Surprisingly, even though the size of the humeral head increases, the slope of the mineral gradient does not change significantly between P7 and P56 (as illustrated by the slope of the Raman data in Fig. 3A, which is plotted in Fig. 3B). This suggests that the mineral gradient is a consistent feature of the mineralizing growth front in mice, agreeing with the interpretation that it arises from an intrinsic tens-of-nanometer scale surface roughness of the interface. Gradients in mineralization levels have also been observed across remodeling osteons, periosteal growth fronts, and cartilage-bone interfaces in several different animal models [31], [40], [41], [42]. Our previous observations in rats have indicated a wider gradient [10]. Further analyses are necessary to explore how the gradient region may scale in larger organisms. Developmentally, it is probable that enthesis mineralization also coincides with mineralization of the proximal epiphysis in larger organisms.

In the present study, throughout the time course investigated, the mineral gradient is maintained as the organic matrix is remodeled from epiphyseal cartilage with disorganized type II collagen fibers to fibrocartilage with more aligned and increased amounts of type I collagen [18]. The zone of gradation shifts radially over time from the center of the humeral head in the early post-natal period to the perimeter of the humeral head by P14 (Fig. 2). The ∼25 µm width of the gradient region in the mouse supraspinatus enthesis is similar to the diameter of the large hypertrophic chondrocytes present early in development [43]. At the early developmental time points P7 and P10, the gradient region was often located between cells (but not always radially outward from cells), resulting in mineral on one side of the cell only (Figure 1B). This observation indicates two possible cell-mediated mineralization mechanisms to produce a graded interface: 1) polarized cells located directly at the interface control mineralization, so that mineralization can occur on one side of the cell but not the other, or 2) matrix mineralization is driven by cells located away from the interface, and the matrix composition itself determines where and how much mineral is deposited. The latter could occur through control of mass transport of Ca and the concentration of mineralization inhibitors and nucleators. An important question is how this process extends to larger organisms. It is unknown how the mineral gradient scales with organism size. Wopenka *et al.* found a slightly wider gradient region (∼100 µm) in rats [10] compared to 25 µm in this study, but it is unknown how and if these results scale for larger organisms. Hypertrophic cell size varies with the longitudinal growth rate; a function of anatomic location and species during development [44], but cell size in mature fibrocartilage is approximately constant across species. The latter observation implies that if the gradient width also scales with size, the gradient zone may be wider than one cell diameter in larger organisms. The relationship between the dimensions of the gradient region and cell size also has important implications for the local stress environment within the joint, as discussed below.

The maximum relative mineral concentration of the mineralized tissue adjacent to the gradient region (measured by Raman at a volumetric scale of several µm^3^ at three spots within the fully mineralized enthesis fibrocartilage) did not vary throughout postnatal development. This result is consistent with other reports from murine calvaria and cortical bone [45], [46]. Bone mineral density of the mineralized tissue at the insertion (measured at a volumetric scale of ∼0.1 mm^3^ by microcomputed tomography), however, increased with age. The area fraction of extracellular matrix relative to cell area (measured for an area of ∼200×50 µm by histomorphometry) increased with time. At early time points, the humeral head tissue consisted of densely packed large chondrocytes separated by thin regions of highly mineralized matrix. These features are not resolvable at the resolution of the micro-computed tomography measurements. As the insertion matured, the cell area fraction decreased relative to the amount of mineralized matrix present, resulting in a higher bulk mineral density. Apparent increases in mineral mass are likely due to structural changes (i.e., increased matrix to cell ratio) or changes in the total amount of mineralized matrix present rather than increases in the mineral-to-collagen ratio within the extracellular matrix.

The carbonate content of the apatite crystals that make up the mineralized component increased with animal age. This finding is consistent with other reports [41], [45], [46], [47], but we did not observe a statistically significant corresponding change in mineral crystallinity. These data suggest that, although the gradient in relative mineral-to-collagen concentration is held approximately constant, the composition of the mineral phase changes throughout development as the animal approaches skeletal maturity. One possible explanation for the change in composition is that, during development, mineral is first deposited on a cartilaginous template, then it is subsequently resorbed and remodeled into mature bone or mineralized fibrocartilage [30]. The observed variations in composition and structure of the mineral phase may play an important role in shaping the evolving mechanical environment at the enthesis.

Nano/micro-characterization by TEM revealed that the morphology of mineralization at the tendon enthesis varied throughout development; although a gradual increase in the mineral-to-collagen content was observed from tendon to bone, consistent with the Raman spectroscopic observations, there were also spatial and temporal variations. The organization of the organic component of the matrix progressed throughout development from a more disorganized matrix characteristic of cartilage [48], [49] to a more ordered matrix with well-aligned fibrils. During development of the enthesis, the morphology of the mineralization front changed from large (∼1 µm) dendritic patches of mineral within the cartilage-like matrix (Fig. 6A, P10) through an intermediate stage with some dendritic-like mineralized patches and also patches with a morphology that appears to match the sparsely organized collagen fiber patterns (Fig. 6B, P14) to a morphology in which mineral appeared to be controlled by well-aligned collagen fibrils (Fig. 6D, P56). Furthermore, significant surface roughness at the tendon-to-bone interface was observed at the nanometer-scale (Figure 7) and this surface roughness could account for the micrometer-scale gradient in mineral to collagen content observed by Raman spectroscopy. Our multi-scale imaging results indicate that markedly different nanometer-scale morphologies may appear as similar mineralization patterns when observed at the micrometer-scale. These results highlight the importance of studying this system across multiple length scales.

The unique morphology and mineral distribution at multiple hierarchical levels have important implications for the mechanics of the tendon-to-bone attachment. A gradient in mineral content at the interface leads to a more gradual increase in tissue stiffness [6], [41], [50], [51], ultimately modulating the strains experienced by cells encapsulated in the matrix. Mechanical cues are critical to the modulation of skeletal patterns and the advancement of growth fronts [52], [53]. The morphology of the tendon-to-bone interface during early postnatal development is very different from that in the mature insertion, as illustrated in Figure 6; however, a mineral gradient is present in both cases. It is possible that the mineral gradient provides different mechanical functions over the time course of murine development. Early in development, the high cell-to-matrix ratio coupled with localized high levels of mineral may lead to enhanced local stresses around the compliant chondrocytes. At this early phase of development, these stresses may serve a mechanotransduction role for the initiation of mineralization. In contrast, growth and maturation produce a decrease in cell-to-matrix ratio, in which the mineral gradient may serve to minimize micrometer-scale stress concentrations at the interface.

To summarize, we observed a gradient in mineral-to-matrix ratio across 20 µm at the mineralization front nearest to the supraspinatus tendon insertion throughout postnatal development. Although mice become much larger over the course of development, the size of the region over which this gradual increase occurred was constant from P7 onwards. This constancy in the width of the gradient region occurred despite temporal variations in matrix structure (evaluated by TEM), cell area fraction relative to matrix (evaluated by optical microscopy), and composition of the mineral crystallites (evaluated by Raman spectroscopy). The mineral concentration of fully mineralized collagen was shown to be constant throughout development. The tissue-level volume fraction of mineral increased throughout development, likely due to a reduction in the volume fraction of cells rather than an increase in mineral density of the extracellular matrix. We believe that these conserved and time-varying aspects of the enthesis have important mechanical implications for the way that the tendon-to-bone attachment grows and maintains stability throughout development.
